# Gastroesophageal Reflux Disease

**DOI:** 10.1155/2013/760750

**Published:** 2013-06-18

**Authors:** Gaurav V. Kulkarni, Fernando A. M. Herbella, P. Marco Fisichella

**Affiliations:** ^1^Department of Surgery, Stritch School of Medicine, Loyola University Medical Center, 2160 South First Avenue, Room 3226, Maywood, IL 60153, USA; ^2^Department of Surgery, Escola Paulista de Medicina, Federal University of Sao Paulo, Sao Paulo, SP, Brazil; ^3^Swallowing Center, Department of Surgery, Stritch School of Medicine, Loyola University Medical Center, 2160 South First Avenue, Room 3226, Maywood, IL 60153, USA

In United States, gastroesophageal reflux disease (GERD) affects almost 20% of the population, and its incidence seems to be rising in relation to the widespread epidemic of obesity. The incidence of GERD, however, is different in other areas of the globe, and there have been multiple recent studies from other developed countries suggesting a correlation between the western lifestyle and emergence of this disease. Moreover, in recent years, diagnostic modalities of more complex manifestations of GERD, such as extraesophageal reflux, have been subjected to refinements. This paper discusses recent research regarding the changing spectrum of GERD in areas of the European Union and Asia subjected to urbanization in recent times and discusses recent and clinically important developments in the diagnostic modalities of extraesophageal reflux.

First Dr. L. Çela et al. from Albania present how westernization is taking its toll with respect to lifestyle changes occurring in regions of Albania where the traditional practices are being replaced by the urban lifestyle and the hazards that come in its wake. Traditionally, Albania has been mentioned as one of the countries, which resisted and refrained from adopting an unhealthy lifestyle due to various regional and economic factors. However, this recent study challenges this notion, as it found that the overall prevalence of GERD was 11.9%. The authors also found no significant sex differences but a higher prevalence of GERD among the older participants, as well as a positive relationship with smoking, physical inactivity, fried food consumption, and obesity. 

A paper by Dr. P. Wu et al. from China similarly showed that foods implicated in the increased risk of GERD are all a major part of the “fast-food” diet of the modern civilization and seem to be making their way into the diet of this ethnic population and causing similar problems as they have in the western world. Specifically, the authors have shown that high intake of meat, oils, salt, and calcium is associated with an increased risk for Reflux Esophagitis (RE) while high intake of protein, carbohydrate, calories from protein, vitamin C, grains and potatoes, fruits, and eggs correlates with a reduced risk for RE. Though this study had some language limitations with the ethnic population, the authors have attempted to standardize the responses of participants for broader comparisons. 

Dr. Y. Chen et al. from Tongji University in Shanghai describe the dual use of SF-36 questionnaire and the rabeprazole test for diagnosis of GERD. A rise to 65 points on the questionnaire in a week from starting 20 mg of rabeprazole per day in the affected and the control groups seemed to be a very reliable, sensitive, and cost efficient method for diagnosing GERD. Independent use of the two methods was associated with a low diagnostic value. 

Dr. K. Zelenik et al. from the Czech Republic have evaluated extraesophageal reflux (EER) using reflux area index, number of reflux, and acid exposure times to assess for response to proton pump inhibitors (PPIs). They found a direct positive correlation between the response and higher incidence of the reflux parameters, which was more pronounced when acid exposure times or reflux area indices were used for diagnosing EER rather than the number of reflux episodes. This study is clinically important since it indicates the relief response that PPIs provide in management of EER that has been proven by standardized testing.

Dr. Ö. I. Emilsson et al. address the issue of different biomarkers for GERD in respiratory diseases by comparing the efficacy of available tests assessing biochemical profiles of patients. It appears that lipid laden macrophage indices and quantification of pepsin do not appear to be sensitive for drawing conclusions regarding the occurrence or severity of GERD due to confounding factors present in pulmonary milieu. Moreover, the success of using bronchoalveolar lavage samples in transplant patients for assessing reflux-induced inflammation cannot be reproduced in other healthier patient populations in view of the invasiveness of the procedure. Recognizing a set of biomarkers rather than any one specific element from the samples obtained by exhaled breath condensates and studying particles in exhaled air have shown promise in initial studies and hold the key to further research. 

Finally, Dr. R. Illig et al. from Austria have proposed that the probability to find one single specific biomarker providing all diagnostic, predictive, and prognostic significance in GERD, Barrett's esophagus, and/or esophageal adenocarcinoma is rather utopian. A panel of more sensitive and specific biomarkers is upcoming and is based on developments in technologies such as RNA and DNA microarrays, methylation profiling, epigenetics, and proteomics in association with bioinformatics. These technologies are promising in providing future insights in the complex GERD-Barrett's esophagus-adenocarcinoma sequence.

In summary, the collection of papers presented in this special issue aims to provide an ample overview of the recent epidemiologic research regarding the geographically changing spectrum of GERD and to illustrate developments in the diagnostic modalities of extraesophageal reflux, with the goal to present the reader with an updated overview of important research in the field of GERD.

*Gaurav V. Kulkarni* *Gaurav V. Kulkarni* *Fernando A. M. Herbella* *Fernando A. M. Herbella* *P. Marco Fisichella* *P. Marco Fisichella*

